# Postoperative complications and hospital costs following open radical cystectomy: A retrospective study

**DOI:** 10.1371/journal.pone.0282324

**Published:** 2023-02-24

**Authors:** Laurence Weinberg, Sarah Aishah Azlina Aitken, Peter Kaldas, Luke Fletcher, Patryck Lloyd-Donald, Peter Le, Daniel Do, Carla Borg Caruana, Dominic Walpole, Joseph Ischia, Ronald Ma, Chong Oon Tan, Dong-Kyu Lee

**Affiliations:** 1 Department of Anesthesia, Austin Health, Heidelberg, Australia; 2 Department of Surgery, The University of Melbourne, Austin Health, Heidelberg, Australia; 3 Department of Critical Care, The University of Melbourne, Austin Health, Heidelberg, Australia; 4 Data Analytics Research and Evaluation (DARE) Centre, Austin Health, Heidelberg, Australia; 5 Business Intelligence Unit, Austin Health, Heidelberg, Australia; 6 Department of Anesthesiology and Pain Medicine, Dongguk University Ilsan Hospital, Goyang, Republic of Korea; Asan Medical Center, University of Ulsan College of Medicine, REPUBLIC OF KOREA

## Abstract

**Objectives:**

To evaluate primarily the relationship between postoperative complications and hospital costs, and secondarily the relationship between postoperative complications and mortality, following radical cystectomy.

**Methods:**

Postoperative complications were retrospectively examined for 147 patients undergoing radical cystectomy at a university hospital between January 2012 and July 2021. Complications were defined and graded using the Clavien–Dindo classification system. In-hospital cost was calculated using an activity-based costing methodology. Regression modelling was used to investigate the relationships among a priori selected perioperative variables, complications, and costs. The effect of complications on postoperative mortality was ascertained using time-dependent coefficients in a Cox proportional hazards regression model.

**Results:**

135 (92%) patients experienced one or more postoperative complications. The medians of hospital cost for patients who experienced no complications and those who experienced complications were $42,796.3 (29,222.9–53,532.5) and $81,050.1 (49,614.8–122,533.6) respectively, *p* < 0.001. Hospital costs were strongly associated with complication severity: Clavien-Dindo grade II complications increased costs by 45.2% (p < 0.001, 95% CI 19.1%–76.6%), and Clavien-Dindo grade III to V complications increased costs by 107.5% (p < 0.001, 95% CI 52.4%–181.8%). Each additional count of complication and increase in Clavien-Dindo complication grade increased the risk of mortality 1.28-fold (RR = 1.28, p = 0.006, 95% CI 1.08–1.53) and 2.50-fold (RR = 2.50, p = 0.012 95% CI 1.23–5.07) respectively.

**Conclusions:**

These findings demonstrate a high prevalence of complications following cystectomy and significant associated increases in hospital costs and mortality. Postoperative complications are a key target for cost-containment strategies.

**Trial registration:**

Trial Registration: Australian New Zealand Clinical Trials Registry (ACTRN:12622000057785.

## Introduction

Bladder cancer is the fourth most diagnosed cancer in males in the US [1] and globally accounted for an estimated 2.9% of cancer deaths in 2020 [2]. While trends in prevalence and mortality in bladder cancer vary geographically [3], the 5-year median survival for patients with regional disease is less than 50% without definitive treatment [4]. Radical cystectomy (RC) is now considered the gold standard for muscle-invasive bladder cancer [5]. With this definitive approach, the 5-year median survival increases by 15%–30% [6]. However, RC carries a high risk of postoperative complications [7–11], which in turn can contribute substantially to healthcare cost [12,13].

Economic growth is currently being outpaced by increases in healthcare expenditure [14]. Therefore, understanding the cost breakdown of postoperative complications associated with RC will enable the use of targeted preventative strategies to improve patient outcomes and reduce expenditure. At present, there have been few studies investigating the association between hospital costs after RC and both the number and severity of postoperative complications [8,10]. Further, existing studies have relied solely on hospital administrative coding data to determine the number and severity of complications without accessing patient records to confirm the accuracy of these data.

Consequently, to address this gap in the literature, we conducted a retrospective cost analysis with the primary aim being to evaluate the relationship between hospital costs and the number and severity of postoperative complications in patients undergoing RC. We hypothesised that an increase in the number and severity of complications would be associated with an increase in hospital costs. Our secondary aim was to examine the association between the development of postoperative complications and mortality.

## Methods

### Study design

We conducted a single-centre retrospective cohort study to better understand postoperative complications following RC and their associated costs. The Austin Health Human Research Ethics Committee approved this study and provided a waiver for participant consent (HREC/19/Austin/88). The study protocol was registered in the Australian New Zealand Clinical Trials Registry (ACTRN:12622000057785). This study is reported following the Strengthening the Reporting of Cohort Studies in Surgery guidelines [15]. This study was conducted at Austin Health, a public university teaching hospital in Australia.

Adult patients aged 18 years or older who underwent elective open or laparoscopic RC between January 2012 and July 2021 were included. We excluded patients who had undergone partial cystectomy or if their cystectomy was secondary to another major procedure (e.g., pelvic exenteration or secondary to trauma).

### Data sources

The collected perioperative data included patient demographics, baseline laboratory variables, smoking history within 1 year of surgery, the American Society of Anesthesiologists (ASA) score [16], the age-adjusted Charlson Comorbidity Index (CCI) [17] and diagnoses of malignancy.

Collected intraoperative data included operation urgency, surgical technique and operative time. The collected postoperative data included intensive care unit (ICU) admission, ICU length of stay, length of hospital stay, destination following discharge, 30-day readmission, and postoperative haemoglobin concentration. Data related to preoperative, intraoperative, and postoperative allogeneic red blood cell transfusion were also collected.

In-hospital death was coded as a Clavien-Dindo (CVD) Grade V surgical complication, and any observed mortality was recorded after surgery until the last observation day of 31 October 2021. Survival days were counted from the surgery start date to the last date of follow-up or the date of death. In the case of follow-up loss before 31 October 2021, the last recorded date was considered the last observation day, and the corresponding cases were treated as censored cases.

### Definitions

Total hospital cost was defined as the sum of direct and indirect in-hospital costs of index admission for RC. Raw costing data were obtained from our institution’s business intelligence unit and costing centre. This data included patient care activities relating to anaesthesia, operative theatre, ICU admission, ward, medical consultations, allied health, pathology, blood products, pharmacy, radiology, medical emergency team calls, and hospital-in-the-home. Costs incurred during the preoperative period were excluded from data analysis to prevent potential confounding due to preoperative cost drivers. In-hospital costs arising from any unplanned readmissions within 30 days of discharge were added to the total cost. Costs were inflated to 31 August 2021 based on the most contemporary Australian consumer price index and converted to US dollars based on the market rate on 31 August 2021. In-hospital costs were calculated according to an activity-based costing methodology that allocated costs based on service volume.

Postoperative complications were defined by the European Perioperative Clinical Outcome definitions [18]. The definition for each specific complication is presented in the (see S1 File). The severity of complications was graded according to the CVD system [19]. Patients were grouped by the most severe complication recorded. Postoperative complications were independently cross-checked with a complete chart review by two authors. The length of hospital stay was defined as the number of days from completion of surgery to discharge. Readmissions were defined as any unplanned readmission 30 days post-discharge. Inpatient mortality was defined according to the definition of CVD Grade V (i.e., death of a patient).

### Statistical analysis

Statistical analysis was performed using IBM SPSS Statistics for Windows, version 23 (IBM Corp., 2015, Armonk, NY, USA) and R, version 4.1.2 (R Development Core Team, Vienna, Austria, 2021). Missing data analysis was performed to detect any variables with a missing rate greater than 5%. In case of a missing rate greater than 5%, multiple imputations were applied to the corresponding variable if the missing patterns were completely at random. For variables with a missing rate of less than 5%, cases were excluded during analysis.

All continuous variables were tested for normality using a quantile-quantile plot. When a variable violated the normality assumption, a variable transformation was applied to re-evaluate normality, or non-parametric statistical methods were considered. The Wilcoxon rank-sum test and Kruskal–Wallis one-way analysis of variance on ranks were used to evaluate the unadjusted hospital cost analysis. In addition, Dunn’s multiple pairwise comparison test was used as a post-hoc test.

To analyse adjusted hospital costs, either Pearson’s or Spearman’s correlation analysis was performed (according to the characteristics of the variables and the results of assumption tests) to evaluate the relationship between hospital cost and pre-, intra-, and postoperative variables. Linear regression modelling was used to investigate the effects of postoperative complications on hospital costs. The autocorrelation of hospital cost was evaluated using Durbin–Watson statistics. Multicollinearity between covariates was tested with variance inflation factor and collinearity diagnostics using eigenvalues. Homoscedasticity was assessed using residual plots.

Mortality in the cystectomy cohort was evaluated using a Kaplan–Meier survival curve for the observed period. Mortality-related variables were assessed using Pearson’s or Spearman’s correlation. The adjusted effect of complications on postoperative mortality was ascertained using time-dependent coefficients in a Cox proportional hazards regression model. The constant hazard ratio assumption was evaluated with Schoenfeld’s residuals test.

The results below are presented as mean ± standard deviation or median (interquartile range) for continuous variables and number (percentage) for categorical variables. Comparative results are presented with a *p* value and corresponding effect size. A two-tailed *p* value below 0.05 was considered statistically significant. Probability values were adjusted by Bonferroni’s correction method as required.

## Results

### Baseline patient characteristics

**Fig 1:**
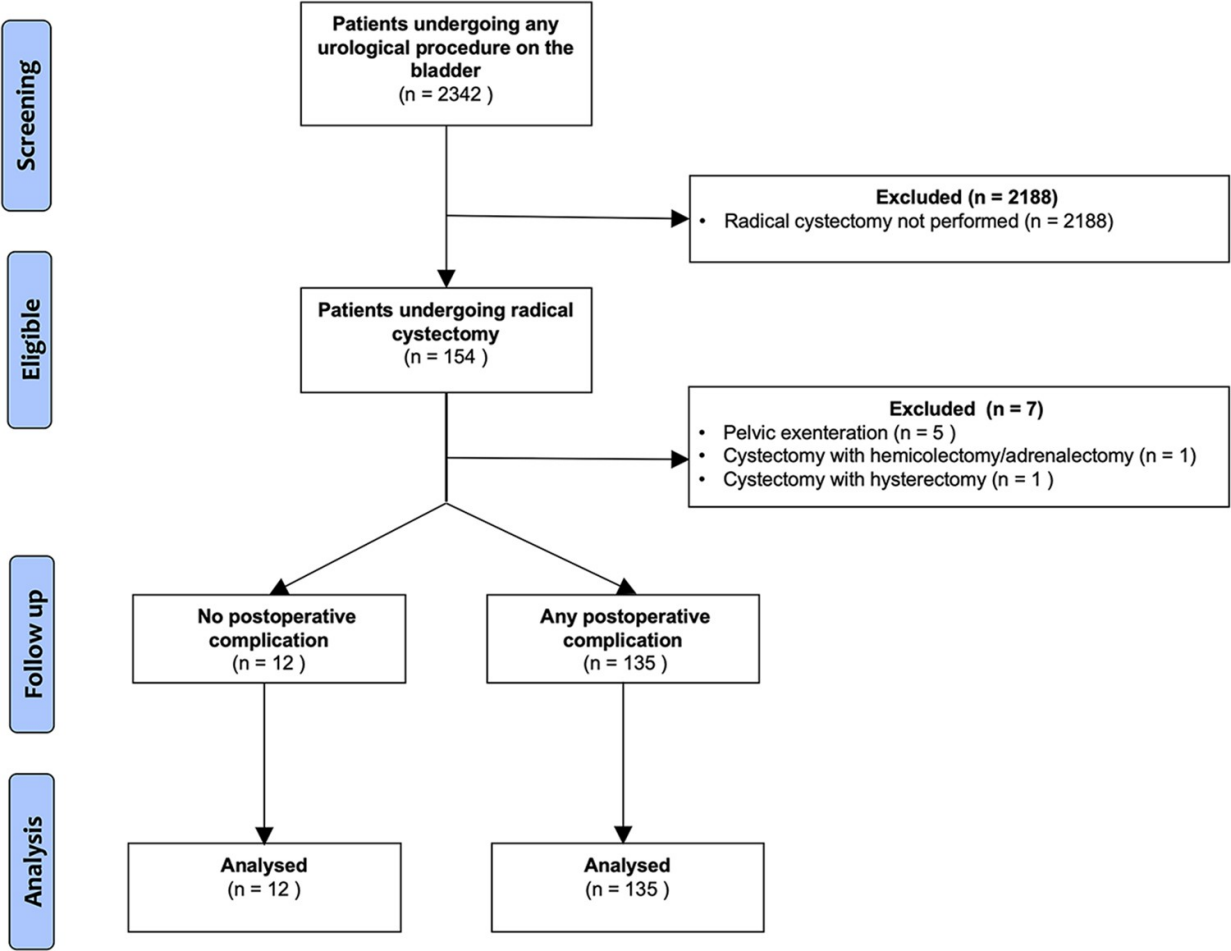
Consort diagram.

From 154 potentially eligible patients who had undergone cystectomy at our institution, seven (4.5%) were excluded (**Fig 1)**. The indication for surgery was malignancy in 131 patients, and non-malignant indications were comprised of neuromuscular bladder dysfunction (*n* = 6), interstitial cystitis (*n* = 4), radiation cystitis (*n* = 3), colo-vesicular fistula (*n* = 1), bladder neck contracture (*n* = 1) and bilateral ureteric strictures (*n* = 1).

The mean age was 63.7 ± 11.6 years, and most patients were male (129 [88%]). Mean height and weight were 172.5 ± 9.2 cm and 81.7 ± 16.6 kg, respectively. Twenty-seven patients (18%) were current smokers, and 103 patients (70%) were married. The median age-adjusted CCI was 6 (5–7). Detailed baseline characteristics and perioperative variables are presented in **Table 1.** The full data sheet is available as a (see S2 File).

**Table 1:** Patients’ characteristics and perioperative variables.

Preoperative		Intraoperative and intensive care		Postoperative	
Variable	Value	Variable	Value	Variable	Value
**Patient characteristics**		**ICU outcomes**		**Postoperative outcomes**	
Age (years)	63.7 ± 11.6	Surgery duration (hr)	6.5 ± 2.5	Postoperative MET calls	46 (31.3)
Weight (kg)	81.7 ± 16.6	Patients requiring ICU care	113 (76.9)	Length of hospital stay (days)	13.1 (9.2–21.2)
Height (cm)	172.5 ± 9.2	ICU length of stay (hr)	16 (12–41.5)	Returned to theatre	11 (7.5)
Sex (male)	129 (87.8)	Patients needing mechanical ventilation	9 (6.1)	**Readmission data**	
Age-adjusted CCI	5.7 ± 2.1, 6 (5–7)			Readmission	112 (76.2)
Smoker within 1 year	129 (87.8)	Mechanical ventilation time (hr)	15 (7–26)	Planned	16 (10.9)
**ASA class**		**Blood results in ICU**		Unexpected	74 (50.3)
I	5 (3.4)	pH, min	7.31 ± 0.05	Not related to surgery	22 (15)
II	45 (30.6)	pH, max	7.42 ± 0.06	**Postoperative blood results**	
III	89 (60.5)	Bicarbonate (mmol/L), min	20.5 ± 2.9	Haemoglobin (g/dL), min	78.2 ± 10.8
IV	8 (5.4)	Lactate (mmol/L), min	0.9 ± 0.4	White cell (× 10^9^/L), max	17.2 ± 5.4
**Preoperative blood results**		Lactate (mmol/L), max	2.3 ± 1.4	Platelet (× 10^9^/L), min	172.9 ± 60.
Haemoglobin (g/L)	131.4 ± 22.4	pCO_2_ (mmHg), min	36.9 ± 5.7	Sodium (mmol/L), min	134.3 ± 3.4
White cell (× 10^9^/L)	7.6 ± 2.8	pCO_2_ (mmHg), max	48 ± 6	Potassium (mmol/L), max	134.3 ± 3.4
Platelet (× 10^9^/L)	263.6 ± 90.7			Chloride (mmol/L), min	5.1 ± 0.6
Sodium (mmol/L)	140.4 ± 3.1			Bicarbonate (mmol/L), min	105.8 ± 3.7
Potassium (mmol/L)	4.4 ± 0.4			Urea (mmol/L), max	20.5 ± 2.9
Chloride (mmol/L)	101.1 ± 3.4			eGFR (mL/min/1.73 m^2^), min	9.5 ± 4.1
Bicarbonate (mmol/L)	25.5 ± 2.7				
Urea (mmol/L)	6.5 ± 2.9				
eGFR (mL/min/1.73 m^2^)	74.9 ± 18.5				

Note. Data are presented as *n* (%); *M* ± *SD*; or *Mdn* (interquartile range). CCI = Charlson Comorbidity Index; ASA = American Society of Anesthesiologists; eGFR = estimated glomerular filtration rate; ICU = intensive care unit; pCO_2_ = partial pressure of carbon dioxide; MET = medical emergency team.

### Postoperative complications

One hundred and thirty-five (92%) patients experienced one or more postoperative complications. In total, 94 (64%) patients had 1–5 complications, and 41 (28%) patients had six or more complications. Regarding the severity of complications, 91 (62%) patients experienced CVD Grade II complications, and 27 (18%) patients experienced a CVD Grade III or IV complication. There was one postoperative death (CVD Grade V). Complications, stratified by each patient according to their worst Clavien–Dindo complication are summarised in **Table 2.**

**Table 2:** Complication incidence, stratified by worst Clavien–Dindo complication grade.

	Worst Clavien-Dindo Complication Grade									
	Number of patients whose worst complication was CVD Grade I N = 16		Number of patients whose worst complication was Grade II N = 91		Number of patients whose worst complication was Grade III N = 14		Number of patients whose worst complication was Grade IV/V N = 14		All patients (with and without complications) N = 147	
**Specific** **Complication**	N (patients who experienced the specific complication outlined in the first column)	%	N (patients who experienced the specific complication outlined in the first column)	%	N (patients who experienced the specific complication outlined in the first column)	%	N (patients who experienced the specific complication outlined in the first column)	%	Number of total patients with a complication	%
** Cardiovascular**										
Myocardial infarction	0	0.0%	0	0.0%	0	0.0%	0	0.0%	0	0.0%
Cardiogenic pulmonary oedema	1	6.3%	6	6.6%	4	28.6%	6	42.9%	17	11.6%
Hypotension (volume depletion or vasoplegia) requiring treatment	5	31.3%	39	42.9%	6	42.9%	12	85.7%	62	42.2%
Brady/tachycardia arrythmia requiring review	0	0.0%	13	14.3%	1	7.1%	5	35.7%	19	12.9%
Other cardiac	0	0.0%	2	2.2%	2	14.3%	0	0.0%	4	2.7%
** Pulmonary**										
Pneumonia	0	0.0%	11	12.1%	1	7.1%	2	14.3%	14	9.5%
Pulmonary embolus	0	0.0%	4	4.4%	1	7.1%	2	14.3%	7	4.8%
Respiratory failure/atelectasis	1	6.3%	5	5.5%	0	0.0%	2	14.3%	8	5.4%
Other pulmonary	0	0.0%	0	0.0%	1	7.1%	1	7.1%	2	1.4%
** Gastrointestinal**										
Ileus/delayed gastric emptying	1	6.3%	57	62.6%	12	85.7%	8	57.1%	78	53.1%
Intra-abdominal collection	0	0.0%	6	6.6%	9	64.3%	3	21.4%	18	12.2%
Surgical site/wound complication	0	0.0%	9	9.9%	2	14.3%	8	57.1%	19	12.9%
Anastomotic leak/breakdown	0	0.0%	5	5.5%	5	35.7%	3	21.4%	13	8.8%
Postoperative haemorrhage	0	0.0%	2	2.2%	2	14.3%	2	14.3%	6	4.1%
Nausea and vomiting	2	12.5%	4	4.4%	1	7.1%	5	35.7%	12	8.2%
Gastrointestinal bleed	0	0.0%	3	3.3%	0	0.0%	1	7.1%	4	2.7%
Other gastrointestinal	0	0.0%	4	4.4%	1	7.1%	3	21.4%	8	5.4%
**Haematalogical**										
Postoperative anaemia	3	18.8%	32	35.2%	10	71.4%	13	92.9%	58	39.5%
Thrombosis	0	0.0%	2	2.2%	1	7.1%	3	21.4%	6	4.1%
** Renal**										
Acute kidney injury	0	0.0%	10	11.0%	3	21.4%	9	64.3%	22	15.0%
Urinary tract infection	0	0.0%	6	6.6%	2	14.3%	2	14.3%	10	6.8%
Other renal	0	0.0%	0	0.0%	1	7.1%	1	7.1%	2	1.4%
** Metabolic**										
Electrolyte derangement	5	31.3%	31	34.1%	13	92.9%	10	71.4%	59	40.1%
Endocrine derangement	0	0.0%	4	4.4%	1	7.1%	1	7.1%	6	4.1%
**Neurological**										
Delirium	2	12.5%	7	7.7%	2	14.3%	6	42.9%	17	11.6%
Postoperative stroke/TIA	0	0.0%	1	1.1%	0	0.0%	1	7.1%	2	1.4%
Other neurological	0	0.0%	2	2.2%	0	0.0%	2	14.3%	4	2.7%
**Infectious**										
Bacteraemia/sepsis	0	0.0%	9	9.9%	5	35.7%	7	50.0%	21	14.3%
**Other**										
Uncontrolled postoperative pain/opioid side effect	0	0.0%	2	2.2%	1	7.1%	1	7.1%	4	2.7%
Dermatological	0	0.0%	1	1.1%	0	0.0%	1	7.1%	2	1.4%
Mechanical fall	0	0.0%	1	1.1%	0	0.0%	0	0.0%	1	0.7%
Syncope	0	0.0%	2	2.2%	0	0.0%	0	0.0%	2	1.4%
Protein-energy undernutrition	0	0.0%	12	13.2%	7	50.0%	6	42.9%	25	17.0%

N = Number of patients who experienced the specific complication outlined in the first column, stratified by the worst Clavien–Dindo complication grade.

% = Percentage of patients who experienced the the specific complication outlined in the first column, stratified by the worst Clavien–Dindo complication grade.

Clinical example:- amongst all patients (N = 147), hypotension occurred in a total of 62 patients (42.2%). The occurrence of hypotension is seen to increase amongst patients that develop more severe complications. Amongst those patients whose worst complication was a Clavien–Dindo grade 1 (N = 16), hypotension occurred in 5 patients (31.3%). Amongst those patients whose worst complication was a Clavien–Dindo grade II (N = 91), hypotension occurred in 39 patients (42.9%). Amongst those patients whose worst complication was a Clavien–Dindo grade III (N = 14), hypotension occurred in 6 patients (42.9%). Amongst those patients whose worst complication was a Clavien–Dindo grade IV/V5 (N = 14), hypotension occurred in 12 patients (85.7%). Note: The sum of N’s in Table 2 is equal to the total number of patients that developed complications, not the total number of complications. In instances where individual complications are grouped together, the complication count will be higher than the number of patients affected. For instance, if a single patient develops both “hypokalaemia” and “hypomagnesaemia”, they will have had two complications, but this will be represented as one occurrence of “electrolyte disorders”.

### Unadjusted hospital cost

**Fig 2:**
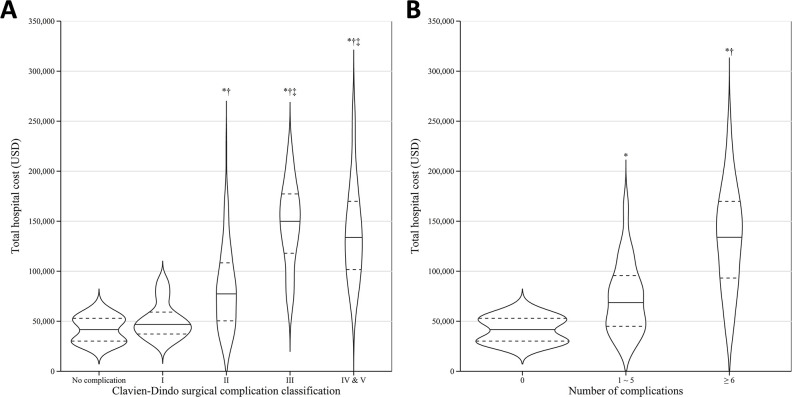
Unadjusted hospital costs categorised by the severity of complications (A) and the number of complications (B). Severity of complications graded by the Clavien–Dindo (CVD) surgical complication classification grades (* vs no complication; † vs CVD Grade I; ‡ vs CVD Grade II). Numbers of complications per patient classified as 0, 1–5 or ≥ 6 complications (* vs no complication; † vs 1–5 complications). Post-hoc Dunn’s test applied after the Kruskal–Wallis one-way analysis of variance on ranks.

Postoperative complications and their associated hospital costs are presented in **Table 3**. The medians of hospital cost for patients who experienced no complications and those who experienced complications were USD 42,796.3 (29,222.9–53,532.5) and USD 81,050.1 (49,614.8–122,533.6), respectively, *p* < 0.001. The median hospital costs according to categorised numbers of complications (i.e., 1–5 or ≥ 6) were statistically different from each other (*p* < 0.001). With regards to complication severity, there was no significant difference between the median costs for patients with no complications and those with CVD Grade I complications. The median hospital cost for CVD Grade II complicated patients was significantly greater than that for patients with no or CVD Grade I complications, and the median costs for CVD Grade III, IV or V patients were significantly higher than those for patients with no, or CVD Grade I or II complications, *p* < 0.00. The unadjusted hospital cost by the categorised number of complications and by the severity of complications is presented in **Fig 2.**

**Table 3:** Postoperative complications and associated hospital costs.

Postoperative complications		Number of patients (%)	Hospital costs (USD) Median IQR
None		12 (8.2)	42,796.3 (29,222.9–53,532.5)
Any		135 (91.8)	81,050.1 (49,614.8–122,533.6)
Number per patient		2 (2–6)	
Grouped	1–5	94 (63.9)	70,679.1 (45,044.7–91,663.3)
	≥ 6	41 (27.9)	134,690.7 (89,919.6–161,608)
CVD grade	I	16 (10.9)	46,316.8 (37,757.0–58,006.9)
	II III IV & V	91 (61.9) 14 (9.5) 14 (9.5)	77,994.3 (48,960.8–107,239.1) 153,189.5 (122,465.4–176,870.2) 133,308.8 (95,745.6–165,320.0)

Note. n = 147. Values are presented as *n* (%) or *Mdn* (interquartile range). CVD grade = Clavien-Dindo surgical complication grade; IQR = interquartile range.

### Adjusted hospital cost

Among the collected variables, coefficients greater than 0.2 or less than -0.2 that were statistically significant were considered as adjusting covariates for the regression model. Although ICU inpatient time and mechanical ventilation time were correlated with postoperative complication number and hospital cost, the variables indicating any postoperative ICU care and mechanical ventilatory assistance during admission were used instead of time variables. Age-adjusted CCI was included as an adjusting covariate due to its clinical significance. The correlation analysis between perioperative variables, postoperative complications and hospital cost is detailed in **S3 File.**

Postoperative maximal sodium concentration and pH were excluded from regression modelling due to multicollinearity. During regression analysis, CVD classifications were grouped into three levels according to the result of unadjusted cost analysis, namely “No complications and CVD I”, “CVD II” and “CVD III to V complications”. The integrated cost-driving effect of complications was not statistically significant, coefficient for having any complications = 1.274, *p* = 0.13, 95% CI [0.93–1.74]. However, having six or more complications significantly increased the hospital costs by 85%, *p* = 0.002, 95% CI [25%–170%]. Hospital costs were also strongly associated with complication severity: CVD Grade II complications increased hospital costs by 45%, *p* < 0.001, 95% CI [19%–77%], and CVD Grade III to V complications increased hospital costs by 110%, *p* < 0.001, 95% CI [52%–180%]. The adjusted hospital cost regression models are detailed in **S3 File.**

### Patients’ survival after cystectomy

**Fig 3:**
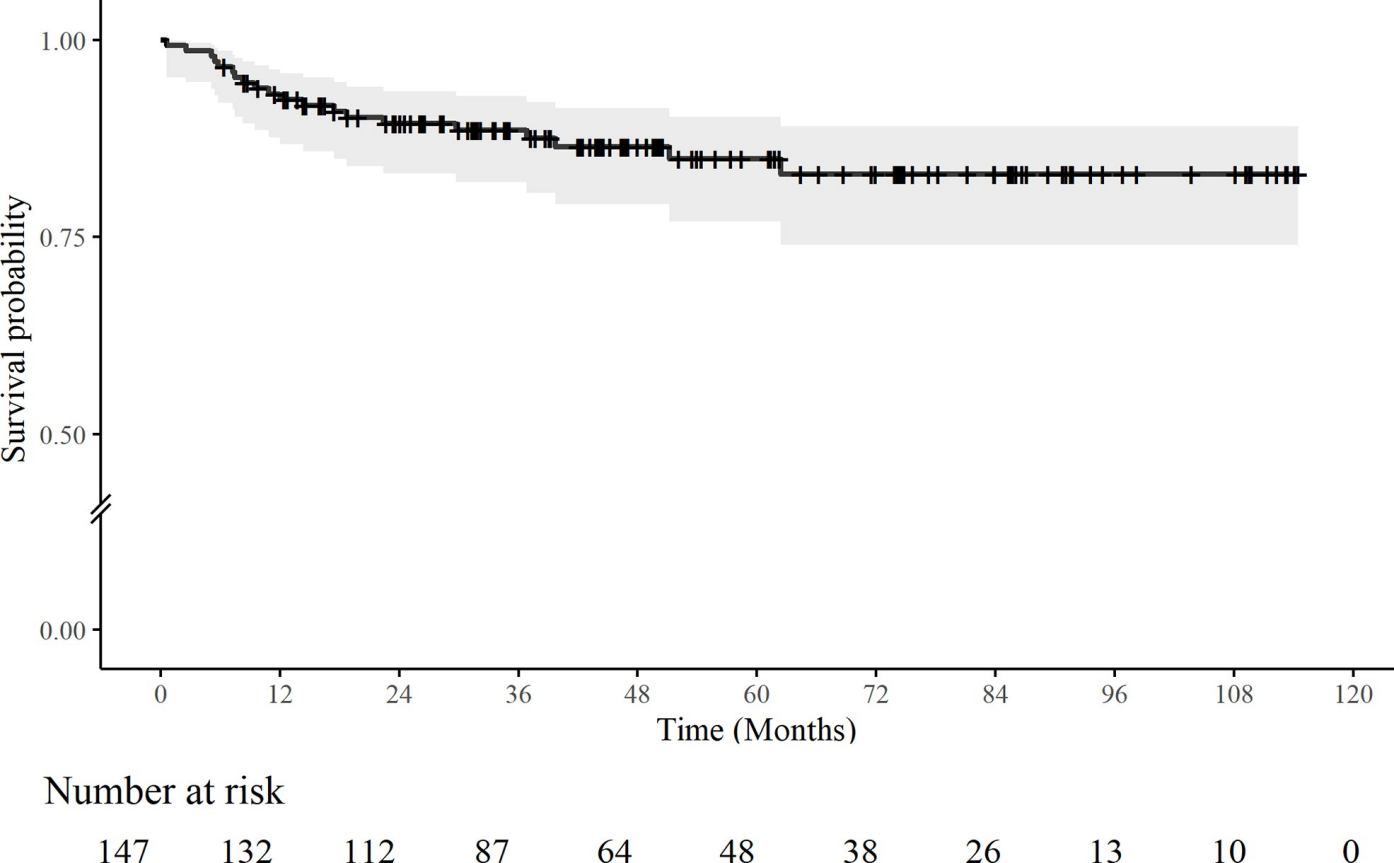
Overall Kaplan-Meier survival curve of cystectomy patients. Any cross marks the censored data at that time point. Shaded areas indicate 95% confidence intervals.

Included patients were observed for a median of 44.1 months (24.9–74.0) after surgery. During this period, 20 patients were deceased, and overall mortality was 14%, 95% CI [8.1%–19%]. One patient died within 90 postoperative days. At one year postoperatively, death was observed in 10 patients (1-year mortality = 6.8%, 95% CI [2.7%–11%]). **Fig 3** shows the overall mortality in this study population.

The relationships between perioperative parameters, complications and mortality are presented in **Table 4.** Only three variables were significantly correlated with mortality, namely age-adjusted CCI, postoperative minimum sodium concentration and readmission. The risk ratio (*RR*) of having any complication was not statistically significant over the whole observation period, *p* = 0.4 and 0.12, before and after postoperative 9 months, respectively. However, each additional number of complications increased the risk of mortality 1.28-fold, *RR* = 1.28, *p* = 0.006, 95% CI [1.08–1.53]. Similarly, each increase in CVD grade of complication increased the risk of mortality 2.50-fold up to postoperative 9 months, *RR* = 2.50, *p* = 0.012 95% CI [1.23–5.07]. After postoperative 9 months, the effect of the number or severity of complications on mortality was non-significant.

**Table 4:** Estimated risk ratios of postoperative mortality by complication presence, number and severity.

Variable		Presence of complications		Number of complications		Severity of complications	
		Risk ratio (95% CI)	*p*	Risk ratio (95% CI)	*p*	Risk ratio (95% CI)	*p*
Age-adjusted CCI		1.33 (1.05–1.70)	0.020*	1.31 (1.03–1.67)	0.029*	1.31 (1.02–1.68)	0.032*
Postoperative minimum sodium concentration		1.23 (1.02–1.47)	0.027*	1.35 (1.11–1.64)	0.003*	1.32 (1.08–1.61)	0.006*
Readmission		6.80 (0.89–51.83)	0.064	7.40 (0.95–57.88)	0.056	6.13 (0.79–47.66)	0.083
Any complication	before POP 9 months	7.18 × 10^7^ (0.00–Inf)	>0.9				
	after POP 9 months	6.14 × 10^7^ (0.00–Inf)	>0.9				
Complications	before POP 9 months			1.28 (1.08–1.53)	0.006*		
	after POP 9 months			1.16 (0.98–1.38)	0.080		
CVD grade	before POP 9 months					2.50 (1.23–5.07)	0.012*
	after POP 9 months					1.78 (0.89–3.56)	0.10

Note. Time-dependent coefficient Cox proportional hazards regression. CI = confidence interval; CCI = Charlson Comorbidity Index; POP = postoperative; CVD grade = Clavien–Dindo surgical complication grade.

## Discussion

In this retrospective cost analysis of patients who had undergone RC, we found that nine out of every 10 patients developed a postoperative complication. In line with our hypothesis, we found that hospital costs increased significantly as postoperative complication count and severity increased. While most complications were minor (i.e., 73% were CVD Grades I or II), the development of a CVD Grade II complication increased hospital costs by 45%. Patients with CVD Grade III–V complications incurred a 110% increase in hospital costs. The development of a single postoperative complication was not associated with an increased mortality risk. However, the mortality risk increased for every additional complication (*RR* = 1.28) and for each increase in CVD grade (*RR* = 2.50) until 9 months post-cystectomy, which is consistent with previous literature [20]. These findings provide an evaluation of the impact of postoperative complications on hospital costs following RC and will allow for targeted and thereby cost-effective strategies for preventing such complications.

Our findings of increased hospital costs in patients with postoperative complications are consistent with those in the previous studies [8,10,12]. In their study, Leow et al. identified postoperative complications as the most significant contributor to hospital costs and found that increased complication severity was a risk factor for high hospital costs [12]. Further studies have similarly observed a positive correlation between hospital costs and the number and severity of complications [8,10]. Our study adds to this body of literature by quantifying the cost increase associated with the increased number and severity of complications.

Interestingly, the incidence of postoperative complications in the present study (92%) was much higher than previously reported figures of 30%–60% [8,11,21]. However, most complications were minor complications (CVD grade I and II). There may be several factors contributing to this high incidence. First, the interpretation of postoperative complications differs depending on the clinician and many papers reporting on the incidence of complications do not report minor complications. We opted for a strict interpretation of the CVD classification system, which resulted in the increase in the total number of complications. Second, we found that some complications were not included in administrative coding, so previous studies relying only on administrative data for billing may have underestimated the incidence of complications. Thirdly, our institution does not perform robotic RC, which a meta-analysis showed to have a significantly lower incidence of postoperative complication rates compared to open RC [22]. However, another study did not find a difference between the incidence of complications in open RC and robotic RC [9].

Understanding the individual cost burden and frequency of complications will enable hospitals to identify where best to target preventative measures. Therefore, future research should include large-scale retrospective cohort studies—stratifying complications by diagnosis, frequency and severity—to determine their individual influence on hospital cost. This, combined with scores to predict complications, such as the recently proposed PT_2_D score [23] could lead to proactive treatments for select individuals at risk of complications. Measures such as this are particularly important, as studies assessing the trend of postoperative complications have found rates to be stable [24,25], even with the introduction of enhanced recovery after surgery [26].

The use of the validated CVD classification system provided us with a robust means to compare the severity of postoperative complications and is widely used within surgical literature. Previous studies have been limited by a reliance on administrative data to identify and grade the severity of complications [8,10,12]. Since treatment received determines complication severity in the CVD system, severity cannot be assessed with great precision using administrative data alone. To address this, we reviewed all patient medical records to ensure that the appropriate classification was assigned and that errors due to administrative coding were minimised. Additionally, being based in one institution was advantageous, as accurate costing information was provided from the hospital’s business intelligence cost unit.

However, our study had several limitations, particularly with respect to the sample size and setting. Our sample of 147 patients is smaller than previous studies, which have had sample sizes up to 23,000 [8]. Additionally, all data were collected from one institution, limiting the generalisability of our results. Finally, at the time this study was conducted, robotic RC was not available at our institution. Previous studies have reported conflicting findings regarding difference between hospital costs and complication rates for robotic-assisted or open RC [9,22]. Nonetheless, this is an important consideration due to the increasing prevalence of robotic RC [27].

## Conclusions

Understanding the financial implications of postoperative complications after RC is important for improving patient care while also keeping health care financially viable. The increase in the number and severity of postoperative complications in this study’s cohort significantly increased their hospital costs and mortality risk. Building on this research will enable hospitals to employ targeted quality-improvement activities to improve patient care and reduce expenditure.

## Supporting information

S1 File Complication definitions. (DOCX)

S2 File Full de-identified data set. (XLSX)

S3 File Correlation analysis and adjusted hospital cost regression models. (DOCX)
