# Supplementary material for: Postoperative complications and hospital costs following open radical cystectomy: A retrospective study
Source: PLoS One. 2023 Feb 24;18(2):e0282324. doi: 10.1371/journal.pone.0282324 (PMC9956632; doi:10.1371/journal.pone.0282324)
Supplement: S1 File — (DOCX) [file pone.0282324.s001.docx]

| **Supplementary file S1 Definitions of complications** | |
| --- | --- |
| **Cardiovascular** | |
| Myocardial infarction | Increase in serum cardiac biomarker values (preferably cardiac troponin) with at least one value above the 99thpercentile upper reference limit and at least one of the following criteria:10 symptoms of ischaemia; new or presumed new significant ST segment or T wave ECG changes or new left bundle branch block; development of pathological Q waves on ECG; radiological or echocardiographic evidence of new loss of viable myocardium or new regional wall motion abnormality; identification of an intracoronary thrombus at angiography or autopsy.  **European Perioperative Clinical Outcome (EPCO) definition.** |
| Cardiogenic pulmonary oedema | Evidence of fluid accumulation in the alveoli due to poor cardiac function.  **European Perioperative Clinical Outcome (EPCO) definition.** |
| Hypotension (volume depletion or vasoplegia) requiring treatment | Mean arterial less 65 mmHg or systolic pressure less than 98 mmHg Reported definitions of intraoperative hypotension in adults undergoing non-cardiac surgery under general anaesthesia. **BMC Anesthesiol. 2022 Mar 11;22(1):69. doi: 10.1186/s12871-022-01605-9. PMID: 35277122** |
| Brady/tachycardia arrythmia requiring review | Electrocardiographic evidence of cardiac rhythm disturbance. **European Perioperative Clinical Outcome (EPCO) definition.** |
| **Pulmonary** | |
| Pneumonia | Two or more serial chest radiographs with at least one of the following (one radiograph is sufficient for patients with no underlying pulmonary or cardiac disease):   - new or progressive and persistent infiltrates - consolidation - cavitation;   at least one of the following:-   - fever (>38°C) with no other recognised cause - leucopaenia (white cell count < 4 × 10^9^/l) or leucocytosis (white cell count >12 × 10^9^/1) - for adults >70 years old, altered mental status with no other recognised cause;   and at least two of the following   - new onset of purulent sputum or change in character of sputum, or increased respiratory secretions, or increased suctioning requirements - new onset or worsening cough, or dyspnoea, or tachypnoea - râles or bronchial breath sounds - worsening gas exchange (hypoxaemia, increased oxygen requirement, increased ventilator demand).   **European Perioperative Clinical Outcome (EPCO) definitions** |
| Pulmonary embolus | A new blood clot or thrombus within the pulmonary arterial system confirmed on co,puted tomography or ventilation perfusion scan  **European Perioperative Clinical Outcome (EPCO) definitions** |
| Respiratory failure/atelectasis | Postoperative PaO2 < 8 kPa (60 mmHg) on room air, a PaO_2_:FI0_2_ ratio <40 kPa (300 mmHg) or arterial oxyhaemoglobin saturation measured with pulse oximetry < 90% and requiring oxygen therapy |
| Other pulmonary | - Pneumothorax: Air in the pleural space with no vascular bed surrounding the visceral pleura - Bronchospasm: Newly detected expiratory wheezing treated with bronchodilators - Aspiration pneumonitis: Acute lung injury after the inhalation of regurgitated gastric contents   **European Perioperative Clinical Outcome (EPCO) definitions** |
| **Gastrointestinal** | |
| Ileus/delayed gastric emptying | Failure to tolerate solid food or defecate for three or more days after surgery |
| Intra-abdominal collection | Ultrasound or computer tomography conformation of a leak of luminal contents from a surgical connection between two hollow viscera. The luminal contents may emerge either through the wound or at the drain site, or they may collect near the anastomosis, causing fever, abscess, septicaemia, metabolic disturbance and/or multiple organ failure.  **European Perioperative Clinical Outcome (EPCO) definitions** |
| Surgical site/wound complication | Superficial incisional surgical site infection as one which meets the following criteria:   - Infection occurs within 30 days after surgery and - Involves only skin and subcutaneous tissue of the incision and - The patient has at least one of the following:   - purulent drainage from the superficial incision   - organisms isolated from an aseptically obtained culture of fluid or tissue from the superficial incision   - at least one of the following symptoms or signs of infection: pain or tenderness, localised swelling, redness or - heat, and superficial incision is deliberately opened by surgeon and is culture positive or not cultured. A culturenegative - finding does not meet this criterion.   - diagnosis of an incisional surgical site infection by a surgeon or attending physician.   Deep incisional surgical site infection as one which meets the following criteria.   - Infection occurs within 30 days after surgery if no implant is left in place or 1 year if implant is in place. - Involves deep soft tissues (e.g. fascial and muscle layers) of the incision. - The patient has at least one of the following:   - purulent drainage from the deep incision but not from the organ/space component of the surgical site   - a deep incision spontaneously dehisces or is deliberately opened by a surgeon and is culture-positive or not - cultured when the patient has at least one of the following symptoms or signs: fever (>388C), or localised pain or - tenderness. A culture-negative finding does not meet this criterion.   - an abscess or other evidence of infection involving the deep incision is found on direct examination, during - surgery, or by histopathological or radiological examination   - diagnosis of an incisional surgical site infection by a surgeon or attending physician. |
| Anastamotic leak/breakdown | Findings on surgical re-exploration or computed tomography confirmation of a leak of luminal contents.  **European Perioperative Clinical Outcome (EPCO) definitions** |
| Postoperative haemorrhage | Postoperative haemorrhage as blood loss within 72 h after the start of surgery resulting in the transfusion of blood or return to theater for surgical exploration |
| Nausea and vomiting | The phenomenon of nausea, vomiting, or retching experienced by a patient in the post-anaesthesia care unit or ward requiring treating with a antiemetic |
| Gastrointestinal bleed | Unambiguous clinical or endoscopic evidence of blood in the gastrointestinal tract. Upper gastrointestinal bleeding (or haemorrhage) is that originating proximal to the ligament of Treitz, in practice from the oesophagus, stomach and duodenum. Lower gastrointestinal bleeding is that originating from the small bowel or colon.  **European Perioperative Clinical Outcome (EPCO) definitions** |
| Other gastrointestinal | Suspected peptic ulcer disease requiring treatment with proton pump inhibitor or conformed with upper gastrointestinal endoscopy |
| **Haematalogical** | |
| Postoperative anaemia | Haemoglobin (Hb) levels <12.0 g/dL in women and men |
| Thrombosis | A new blood clot or thrombus within the venous or arterial system system.  **European Perioperative Clinical Outcome (EPCO) definitions** |
| **Renal** | |
| Acute kidney injury | Stage 1: 1.5–1.9 times baseline value within 7 days for or >27 umol/l increase within 48 h  Stage 2: 2.0–2.9 times baseline value within 7 days  Stage 3: 3.0 times baseline within 7 days or increase in serum creatinine to 354 umol /l (with an acute rise of > 44 umol l/1)  **Kidney Disease Improving Global Outcomes (KDIGO) definition** |
| Urinary tract infection | A positive urine culture of >10^5^ colony forming units/ml with no more than two species of micro-organisms, and with at least one of the following symptoms or signs: fever (>38^8^C), urgency, frequency, dysuria, suprapubic tenderness, costovertebral angle pain or tenderness with no other recognised cause.  **European Perioperative Clinical Outcome (EPCO) definitions** |
| Other renal | Haematuria requiring bladder wash out |
| **Metabolic** | |
| Electrolyte derangement | Electrolyte abnormality outside of laboratory normal refence range requiring pharmacological intervention or treatment |
| Endocrine derangement | Hypoglycemia <3.9 mmol/L requiring pharmacological intervention with intravenous dextrose or glucose  Hyperglycemia: blood glucose >10 mmol/l requiring subcutaneous (SC) rapid-acting insulin analogs or with an intravenous infusion of regular insulin. |
| **Neurological** | |
| Delirium/Hallucinations | Documented definition of delirium in the medical records defined by the Intensive Care Delirium Screening Checklist, CAM-ICU assessment tool, or DSM-V criteria for diagnosis. |
| Postoperative stroke/TIA | The ACS-NSQIP definition: an embolic, thrombotic or haemorrhagic cerebral event with persistent residual motor, sensory or cognitive dysfunction (e.g., hemiplegia, hemiparesis, aphasia, sensory deficit, impaired memory). |
| Other neurological | Post-surgical neuropathy attributed to mechanical factors, such as compression, stretch, or contusion |
| **Infectious** | |
| Bacteraemia/sepsis | CDC definition of a laboratory confirmed bloodstream infection as one which meets at least one of the following criteria which should not be related to infection at another site:   - Patient has a recognised pathogen cultured from one or more blood cultures and the organism cultured from blood is not related to an infection at another site - Patient has at least one of the following signs or symptoms: fever >38°C, chills or hypotension, and at least one of the following:   - common skin contaminant cultured from two or more blood cultures drawn on separate occasions   - common skin contaminant cultured from at least one blood culture from a patient with an intravascular line, and the physician institutes appropriate antimicrobial therapy   - Ppositive blood antigen test.   **European Perioperative Clinical Outcome (EPCO) definitions** |
| **Other** | |
| Uncontrolled postoperative pain/opioid side effect | Severe pain > 7 based the 11-point numeric scale ranging from '0' representing one pain extreme (e.g., “no pain”) to '10' representing the other pain extreme (e.g. “pain as bad as you can imagine” or “worst pain imaginable”). |
| Dermatological | New onset skin rash |
| Mechanical fall | A fall secondary to an external force or object that led to the fall |
| Syncope | Documented history of fainting or passing out caused by a temporary drop in the amount of blood that flows to the brain |
| Protein-energy undernutrition | Documented diagnosis in medical record by qualified dietician based on energy deficit due to deficiency of all macronutrients and micronutrients due to inadequate nutrient intake or secondary to disorders or drugs that interfere with nutrient use.  Diagnosis based on history, body mass index, serum albumin, total lymphocyte count, CD4+ count, serum transferrin. Diagnosis often accompanied by complete blood count, electrolytes, blood urea nitrogen, glucose, calcium, magnesium, and phosphate |
