# Supplementary material for: Postoperative complications and hospital costs following open radical cystectomy: A retrospective study
Source: PLoS One. 2023 Feb 24;18(2):e0282324. doi: 10.1371/journal.pone.0282324 (PMC9956632; doi:10.1371/journal.pone.0282324)
Supplement: S3 File — (DOCX) [file pone.0282324.s003.docx]

Supplementary file S3 Table 1. Correlation analysis between perioperative variables, and postoperative complications and hospital cost

| **Variable** | **Any complications** | | **Number of complications** | | **CVD grade** | | **Hospital cost^‡^** | | **Mortality** | |
| --- | --- | --- | --- | --- | --- | --- | --- | --- | --- | --- |
|  | **Coefficient** | ***p*** | **Coefficient** | ***p*** | **Coefficient** | ***p*** | **Coefficient** | ***p*** | **Coefficient** | ***p*** |
| Age | –0.057 | 0.494 | –0.137 | 0.098 | –0.095 | 0.253 | –0.128 | 0.123 | 0.127 | 0.125 |
| Weight | –0.138 | 0.094 | –0.071 | 0.395 | 0.005 | 0.952 | –0.028 | 0.737 | –0.030 | 0.717 |
| Height | –0.079 | 0.343 | –0.094 | 0.258 | 0.009 | 0.918 | –0.157 | 0.057 | –0.029 | 0.738 |
| Sex (male) | –0.036 | 0.669 | 0.103 | 0.213 | 0.158 | 0.056 | –0.001 | 0.991 | 0.148 | 0.073 |
| Smoker within 1 year | –0.074 | 0.391 | –0.108 | 0.208 | –0.068 | 0.427 | –0.090 | 0.293 | –0.091 | 0.288 |
| ASA class | –0.113 | 0.174 | –0.046 | 0.584 | –0.024 | 0.775 | –0.025 | 0.762 | 0.009 | 0.915 |
| Age-adjusted CCI | 0.004 | 0.960 | 0.020 | 0.810 | 0.032 | 0.699 | –0.047 | 0.568 | 0.204^†^ | 0.013^*^ |
| Preoperative bloods |  |  |  |  |  |  |  |  |  |  |
| Haemoglobin | –0.282^†^ | 0.001^*^ | –0.172 | 0.042 | –0.166 | 0.049 | –0.079 | 0.349 | –0.075 | 0.376 |
| White cell | –0.099 | 0.242 | 0.023 | 0.788 | 0.057 | 0.500 | 0.027 | 0.749 | –0.018 | 0.832 |
| Platelet | –0.050 | 0.553 | 0.053 | 0.536 | 0.030 | 0.727 | –0.047 | 0.582 | –0.048 | 0.574 |
| Sodium | –0.056 | 0.510 | –0.005 | 0.955 | 0.021 | 0.805 | –0.091 | 0.283 | –0.009 | 0.913 |
| Potassium | –0.057 | 0.501 | –0.053 | 0.535 | –0.002 | 0.981 | –0.046 | 0.589 | 0.096 | 0.260 |
| Chloride | –0.053 | 0.533 | –0.071 | 0.403 | –0.015 | 0.861 | –0.171 | 0.043 | –0.065 | 0.444 |
| Bicarbonate | –0.074 | 0.384 | –0.014 | 0.868 | –0.040 | 0.634 | 0.083 | 0.328 | –0.024 | 0.774 |
| Urea | –0.084 | 0.325 | –0.053 | 0.536 | –0.003 | 0.970 | –0.088 | 0.301 | 0.083 | 0.330 |
| eGFR | 0.038 | 0.658 | 0.054 | 0.528 | 0.029 | 0.731 | 0.107 | 0.207 | –0.137 | 0.104 |
| Patients requiring ICU care | –0.046 | 0.583 | –0.034 | 0.681 | 0.009 | 0.919 | –0.014 | 0.862 | 0.029 | 0.723 |
| ICU length of stay | 0.106 | 0.202 | 0.236^†^ | 0.004^*^ | 0.173 | 0.036 | 0.231^†^ | 0.005^*^ | 0.098 | 0.236 |
| Postoperative mechanical ventilation | 0.076 | 0.359 | 0.270^†^ | 0.001^*^ | 0.248^†^ | 0.002^*^ | 0.099 | 0.233 | 0.147 | 0.076 |
| Mechanical ventilation time | 0.076 | 0.360 | 0.269^†^ | 0.001^*^ | 0.246^†^ | 0.003^*^ | 0.033 | 0.692 | 0.151 | 0.068 |
| Surgery duration | 0.129 | 0.118 | 0.085 | 0.307 | 0.070 | 0.401 | 0.255^†^ | 0.002^*^ | 0.048 | 0.560 |
| Returned to theatre | 0.085 | 0.307 | 0.371^†^ | < 0.001^*^ | 0.341^†^ | < 0.001^*^ | 0.224^†^ | 0.006^*^ | –0.113 | 0.174 |
| Postoperative MET calls | 0.139 | 0.094 | 0.298^†^ | < 0.001^*^ | 0.165 | 0.046 | 0.047 | 0.570 | 0.005 | 0.948 |
| Postoperative bloods |  |  |  |  |  |  |  |  |  |  |
| Haemoglobin, min | –0.113 | 0.174 | –0.223^†^ | 0.007^*^ | –0.236^†^ | 0.004^*^ | –0.236^†^ | 0.004^*^ | 0.027 | 0.744 |
| White cell, max | 0.092 | 0.270 | 0.178 | 0.031 | 0.133 | 0.109 | 0.081 | 0.330 | 0.020 | 0.807 |
| Platelet, min | –0.016 | 0.844 | 0.035 | 0.670 | 0.044 | 0.595 | –0.126 | 0.127 | 0.052 | 0.534 |
| Sodium, min | –0.020 | 0.807 | –0.223^†^ | 0.007^*^ | –0.182 | 0.027 | –0.235^†^ | 0.004^*^ | 0.173 | 0.036^*^ |
| Potassium, max | 0.028 | 0.732 | 0.143 | 0.084 | 0.189 | 0.022 | 0.179 | 0.030 | 0.041 | 0.624 |
| Chloride, min | 0.120 | 0.146 | 0.145 | 0.080 | 0.174 | 0.035 | 0.111 | 0.180 | –0.004 | 0.964 |
| Bicarbonate, min | –0.096 | 0.246 | –0.157 | 0.058 | –0.150 | 0.069 | –0.178 | 0.031 | –0.027 | 0.749 |
| Urea, max | 0.037 | 0.657 | 0.079 | 0.339 | 0.164 | 0.046 | 0.115 | 0.167 | –0.023 | 0.785 |
| eGFR, min | 0.012 | 0.885 | 0.044 | 0.595 | –0.055 | 0.509 | 0.061 | 0.461 | –0.011 | 0.893 |
| Postoperative arterial blood gas |  |  |  |  |  |  |  |  |  |  |
| pH, min | 0.004 | 0.960 | 0.077 | 0.353 | 0.003 | 0.968 | 0.088 | 0.289 | 0.029 | 0.725 |
| pH, max | 0.107 | 0.200 | 0.361^†^ | < 0.001^*^ | 0.234 | 0.004^*^ | 0.306^†^ | < 0.001^*^ | 0.055 | 0.510 |
| pCO_2_, min | –0.176 | 0.034 | –0.253^†^ | 0.002^*^ | –0.165 | 0.046 | –0.255^†^ | 0.002^*^ | –0.063 | 0.452 |
| pCO_2_, max | –0.048 | 0.565 | –0.045 | 0.588 | 0.014 | 0.863 | –0.031 | 0.713 | –0.059 | 0.480 |
| Lactate, min | –0.193 | 0.020 | –0.321^†^ | < 0.001^*^ | –0.283 | 0.001^*^ | –0.144 | 0.085 | –0.091 | 0.278 |
| Lactate, max | –0.015 | 0.855 | 0.063 | 0.451 | 0.065 | 0.437 | 0.038 | 0.646 | –0.065 | 0.437 |
| Length of hospital stay | 0.362^†^ | < 0.001^*^ | 0.700^†^ | < 0.001^*^ | 0.619^†^ | < 0.001^*^ | 0.462^†^ | < 0.001^*^ | 0.123 | 0.136 |
| Readmission | 0.125 | 0.131 | 0.163 | 0.048 | 0.133 | 0.109 | 0.396^†^ | < 0.001^*^ | 0.175 | 0.034^*^ |

*Note*. Values were obtained using either Pearson’s or Spearman’s correlation analysis. CVD grade = Clavien–Dindo surgical complication grade; ASA = American Society of Anesthesiologists; CCI = Charlson Comorbidity Index; eGFR = estimated glomerular filtration rate; ICU = intensive care unit; MET = medical emergency team; pCO_2_ = partial pressure of carbon dioxide.

‡ Log-transformed hospital cost.

† Statistically significant coefficient greater than 0.200 or less than –0.200.

* *p* < 0.166 (complications); *p* < 0.050 (hospital cost and mortality).

Supplementary file S3 Table 2. Adjusted hospital cost regression models

|  | | **Presence of complications** | | **Number of complications** | | **Severity of complications** | |
| --- | --- | --- | --- | --- | --- | --- | --- |
| **Variable** | | ***b*** | ***p*** | ***b*** | ***p*** | ***b*** | ***p*** |
| (Constant) | | 93972.331 (32583.670‒270395.836) | < 0.001* | 82413.812 (29444.216–230674.719) | < 0.001* | 91201.084 (35563.132–233345.806) | < 0.001* |
| Age-adjusted CCI | | 0.989 (0.951‒1.028) | 0.558 | 0.993 (0.957–1.033) | 0.739 | 0.993 (0.957–1.030) | 0.693 |
| Preoperative haemoglobin | | 0.998 (0.995–1.002) | 0.475 | 1.000 (0.995–1.002) | 0.594 | 0.998 (0.995–1.002) | 0.429 |
| Surgery duration | | 1.023 (0.986–1.062) | 0.213 | 1.026 (0.989–1.064) | 0.165 | 1.021 (0.986–1.057) | 0.218 |
| ICU care during admission | | 0.953 (0.782–1.159) | 0.624 | 0.986 (0.815–1.197) | 0.893 | 0.959 (0.798–1.153) | 0.657 |
| Postoperative mechanical ventilator assist | | 1.047 (0.755–1.452) | 0.782 | 0.881 (0.630–1.233) | 0.459 | 0.871 (0.634–1.199) | 0.396 |
| Postoperative MET calls | | 0.959 (0.895–1.028) | 0.240 | 0.946 (0.885–1.014) | 0.115 | 0.964 (0.904–1.03) | 0.281 |
| Returned to theatre | | 1.493 (1.094–2.037) | 0.012* | 1.274 (0.925–1.754) | 0.137 | 1.236 (0.910–1.683) | 0.172 |
| Postoperative haemoglobin, min | | 0.995 (0.986–1.002) | 0.179 | 0.995 (0.986–1.002) | 0.157 | 0.995 (0.989–1.002) | 0.258 |
| pCO_2_, min | | 0.989 (0.973–1.002) | 0.092 | 0.989 (0.975–1.002) | 0.092 | 0.984 (0.973–0.998) | 0.028* |
| Lactate, min | | 0.971 (0.789–1.194) | 0.773 | 1.021 (0.832–1.250) | 0.849 | 1.021 (0.841–1.239) | 0.826 |
| Length of hospital stay | | 1.012 (1.005–1.019) | < 0.001* | 1.007 (1.000–1.014) | 0.091 | 1.005 (0.998–1.014) | 0.134 |
| Readmission | | 1.663 (1.387–2.000) | < 0.001* | 1.581 (1.321–1.897) | < 0.001* | 1.589 (1.340–1.884) | < 0.001* |
| No complications, or CVD grade I and no complications† | | (Reference) |  | (Reference) |  | (Reference) |  |
| Any complications | | 1.274 (0.931–1.738) | 0.129 |  |  |  |  |
| Complications | 1–5 |  |  | 1.300 (0.959–1.758) | 0.090 |  |  |
|  | ≥ 6 |  |  | 1.849 (1.250–2.742) | 0.002* |  |  |
| CVD grade | II |  |  |  |  | 1.452 (1.191–1.766) | < 0.001* |
|  | III–V |  |  |  |  | 2.075 (1.524–2.818) | < 0.001* |
| Model diagnostics | | *R*^2^ = 0.457; Durbin–Watson statistic = 1.948; *F*(0.272, 14) = 7.449, *p*< 0.001 | | *R*^2^ = 0.493; Durbin–Watson statistic = 1.922; *F*(0.274, 15) = 7.980, *p*< 0.001 | | *R*^2^ = 0.536; Durbin–Watson statistic = 2.065; *F*(0.297, 15) = 9.454, *p*< 0.001 | |

*Note*. Regression models were estimated using the log-transformed hospital cost as the dependent variable. The re-transformed coefficients are expressed with their 95% confidence intervals in parentheses. Model diagnostics are discriminated with Bonferroni’s corrected significance level. Postoperative maximal sodium concentration and pH were excluded due to multicollinearity. CCI = Charlson Comorbidity Index; ICU = intensive care unit; pCO_2_ = partial pressure of carbon dioxide; CVD grade = Clavien–Dindo surgical complication grade.

* *p* < 0.050. †: No complication is the reference group for the presence and numbers of complications, CVD grade I or no complications is for the CVD
